# The effect of aldosterone and aldosterone blockade on the progression of chronic kidney disease: a randomized placebo-controlled clinical trial

**DOI:** 10.1038/s41598-020-73638-4

**Published:** 2020-10-06

**Authors:** Hitoshi Minakuchi, Shu Wakino, Hidenori Urai, Arata Kurokochi, Kazuhiro Hasegawa, Takeshi Kanda, Hirobumi Tokuyama, Hiroshi Itoh

**Affiliations:** Department of Internal Medicine, School of Medicine, Keio University, 35 Shinanomachi, Shinjuku-ku, Tokyo, 160-8582 Japan

**Keywords:** Diseases, Nephrology, Risk factors

## Abstract

The progression of chronic kidney disease (CKD) cannot be completely inhibited. We first explored factors contributing to CKD progression in patients with CKD in a prospective observational study. In the next phase, we focused on the effects of aldosterone, conducting a single-blinded placebo-controlled study using the selective mineralocorticoid receptor antagonist (MRA), eplerenone (25 mg/day). We recruited patients with CKD stage 2 and 3 whose plasma aldosterone concentration was above 15 ng/dL based on the prior data of a prospective observational study. In the CKD cohort study (n = 141), baseline plasma aldosterone concentration was identified as an independent contributory factor for the future rate of change in estimated glomerular filtration rate (eGFR). When the cut-off value for aldosterone was set at 14.5 ng/dL, the decline rate was significantly higher in patients with higher plasma aldosterone concentration (− 1.22 ± 0.39 ml/min/1.73 m^2^/year vs. 0.39 ± 0.40 ml/min/1.73 m^2^/year, *p* = 0.0047). In the final intervention study, in the eplerenone group, eGFR dropped at 6 months after the initiation of the study, and thereafter eGFR was maintained until the end of the study. At 24 months and 36 months, eGFR was significantly higher in the eplerenone group than in the placebo group. In conclusion, MRA can be an effective strategy in preventing CKD progression, especially in patients with high plasma aldosterone.

## Introduction

The prevalence of chronic kidney disease (CKD) is increasing mainly as a result of an ongoing epidemic of obesity, metabolic syndrome, and diabetes mellitus^1^. Numerous clinical studies have established a pivotal role for the renin–angiotensin–aldosterone system (RAAS) in the progression of CKD^2–4^, and inhibition of the RAAS with angiotensin-converting enzyme inhibitors (ACEIs) and angiotensin type 1 receptor blockers (ARBs) has become a mainstay in the management of CKD^5^. Despite treatment with ACEIs and ARBs, many patients with CKD have persistent proteinuria, a risk factor for progressive renal failure^6,7^.

Aldosterone and its antagonists have some roles in the progression of renal failure independently of effects on blood pressure. In a rat model, aldosterone contributes to the progression of kidney disease through hemodynamic and direct cellular actions^8^. Blockade of aldosterone receptors by mineralocorticoid receptor antagonists (MRAs), such as spironolactone and eplerenone, results in a significant reduction of proteinuria in patients with many types of kidney diseases^9–12^. Two meta-analyses demonstrated that MRAs effectively reduce proteinuria combined with ACEIs and ARBs^13,14^. However, recently a large-scale clinical trial testing the effects of a selective non-steroidal MRA, finerenone has been finished. The duration of the trial was approximately 5.5 years^15^ and the only diabetic patients were included in the trial. The long-term effects on renal function—that is, on estimated glomerular filtration rate (eGFR)—were examined and the favorable effects by MRA has been announced in press release^16^. However, the studies until this trial were not designed to demonstrate the beneficial impact of MRAs on long-term renal outcome. Most of the studies evaluated the reduction of proteinuria with MRAs for the short duration^17^. Therefore, the data on the long-term effects of MRA on the eGFR decline in CKD, especially non-diabetic CKD patients are still scarce.

We aimed to: (1) explore factors that contribute to CKD progression, (2) elucidate the long-term effects of excessive aldosterone (plasma level above 15 ng/dL) on renal function by prospective observation of patients with CKD, and (3) conduct a placebo-controlled single-blinded intervention trial using an MRA, eplerenone (EPL) to demonstrate renoprotective effects by MRA.

## Results

### Baseline patient characteristics of the prospective observation study

**Figure 1:**
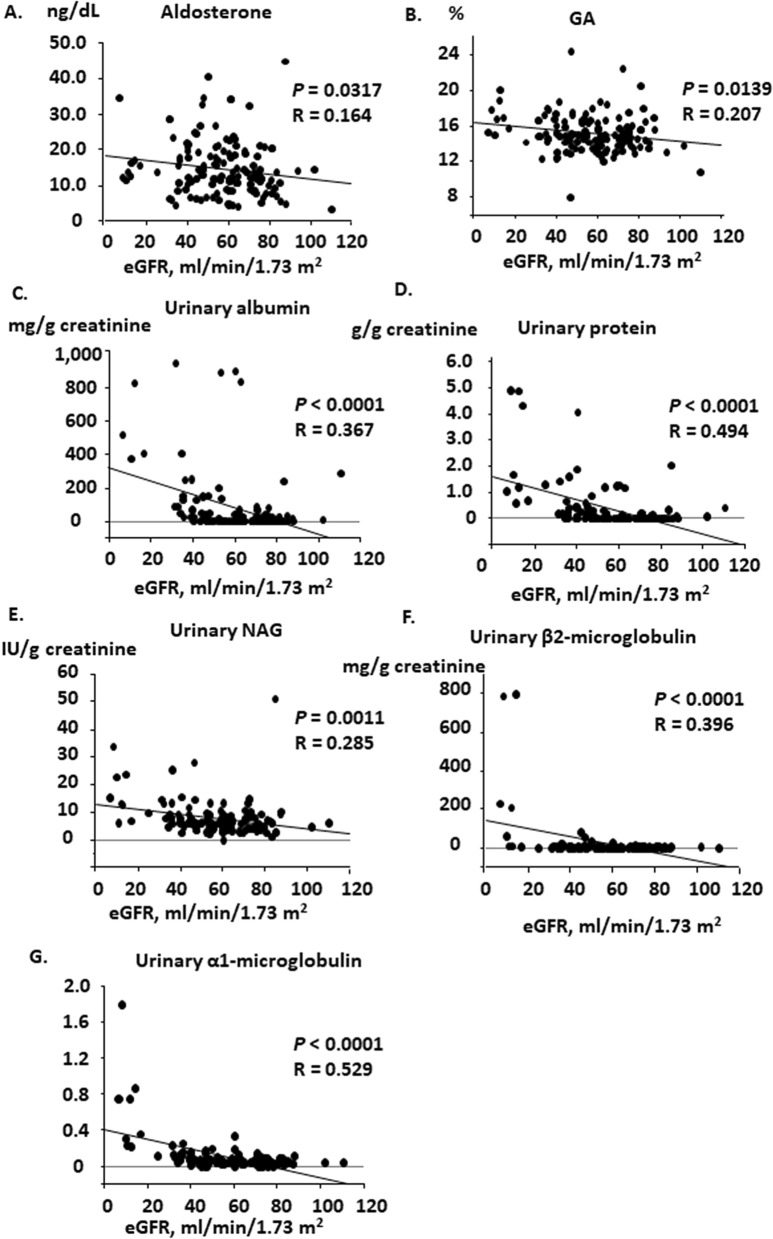
Linear regression analysis between eGFR and parameters in the cross-sectional CKD cohort study. Significant associations were observed between eGFR and the levels of (**A**) plasma aldosterone concentration, (**B**) glycosylated albumin, (**C**) urinary excretion of albumin, (**D**) urinary excretion of protein, (**E**) urinary excretion of N-acetyl-β-D-glucosaminidase, (**F**) urinary excretion of β2-microglobulin, and (G) urinary excretion of α-microglobulin. CKD, chronic kidney disease; eGFR, estimated glomerular filtration rate; GA, glycosylated albumin; NAG, N-acetyl-β-D-glucosaminidase.

For the first phase of the study, we consecutively recruited 151 patients with CKD; their baseline characteristics are described in Table 1. The mean age was 66.8 years and 51.7% were male. The prevalence of hypertension was 89%, and the cause of CKD was: nephrosclerosis (56%), chronic glomerulonephritis (29%), and diabetic nephropathy (14%). Most of the patients took antihypertensive medication; angiotensin II receptor blockade (ARBs) or an ACEI was administered to 59% of the patients. The clinical parameters of the cohort are also shown in Table 2. The systolic and diastolic blood pressures were 133 ± 1.20 mmHg and 75.8 ± 0.8 mmHg as mean ± standard error (SE), respectively. The values of eGFR was 57.2 ± 1.64 ml/min/1.73 m^2^ as mean ± SE. The plasma concentration of active renin was 15.2 ± 1.73 ng/dL, aldosterone 14.8 ± 0.65 ng/dL, and cortisol 12.1 ± 0.29 μg/dL. The association between various parameters and eGFR was examined by linear regression analysis. Plasma aldosterone concentration (PAC) and glycated albumin concentration were associated with eGFR (Fig. 1, aldosterone, R = 0.164, *p* = 0.0317; glycated albumin, R = 0.207, *p* = 0.0139). Urinary excretion of albumin, protein, NAG, β2-microglobulin, and α1-microglobulin were associated with eGFR (Fig. 1, albumin, R = 0.367, *p* < 0.001; protein, R = 0.494, *p* < 0.001; NAG, R = 0.285, *p* = 0.0011; β2-microglobulin, R = 0.396, *p* < 0.0001; α1-microglobulin, R = 0.529, *p* < 0.0001). However, systolic blood pressure (SBP) nor diastolic blood pressure (DBP) was not associated with eGFR (Supplementary Fig. 1A, SBP, *p* = 0.9074; DBP, *p* = 0.2740). Nor was the association detected between PAC and SBP or between PAC and DBP (Supplementary Fig. 1B, *p* = 0.5821 and *p* = 0.8012, respectively).

**Table 1:** Baseline characteristics of the CKD cohort in the cross-sectional study-1.

Number	141
Age, years (oldest, youngest)	66.8 ± 1.6 (88, 16)
Gender, male/female	73/68
**Cause of CKD (%)**	
Nephrosclerosis	79 (56.0%)
Chronic glomerulonephritis	41 (29.1%)
Diabetic nephropathy	20 (14.2%)
Other	1 (0.7%)
**CKD stage (%)**	
G1	3 (2.1%)
G2	68 (48.2%)
G3a	34 (24.1%)
G3b	27 (19.1%)
G4	2 (1.4%)
G5	7 (5.0%)
**Antihypertensive medication (%)**	
ARB/ACEI	84 (59.6%)
Calcium channel blocker	92 (65.2%)
Diuretic	19 (13.5%)
β-blockade	35 (24.8%)
α-blockade	10 (7.1%)
**Other medication (%)**	
Statin	40 (28.4%)
Antiplatelet	32 (22.7%)

Age is expressed as mean ± SEM. ACEI, angiotensin converting enzyme inhibitor; ARB, angiotensin II receptor blocker; CKD, chronic kidney disease; SEM, standard error of the mean.

**Table 2:** Baseline characteristics of the CKD cohort in the cross-sectional study-2.

Parameter	Value (max, min)
SBP, mmHg	133 ± 1.20 (171, 105)
DBP, mmHg	75.8 ± 0.8 (91, 48)
eGFR, ml/min/1.73 m^2^	57.2 ± 1.64 (110, 6.78)
Serum potassium, mEq/l	3.76 ± 0.67 (5.32, 2.84)
Plasma active renin, pg/mL	15.2 ± 1.73 (163, 2.10)
PAC, ng/dL	14.8 ± 0.65 (44.8, 3.2)
Plasma cortisol, μg/dL	12.1 ± 0.29 (21.6, 4.40)
Plasma fasting blood sugar, mg/dL	109 ± 1.33 (197, 72)
Plasma fasting insulin concentration, μU/mL	11.0 ± 0.84 (72, 1.0)
Glycated albumin (%)	15.1 ± 0.18 (24.3, 8.0)
Serum triglyceride, mg/dL	133 ± 7.4 (648, 31)
Serum LDL-cholesterol, mg/dL	118 ± 2.8 (215, 32)
Serum HDL-cholesterol, mg/dL	57.6 ± 1.32 (92, 24)
Urinary protein, g/g creatinine	0.351 ± 0.08 (4.91, 0)
Urinary albumin, mg/g creatinine	81.7 ± 17.9 (950, 0)
Urinary NAG, IU/g creatinine	7.87 ± 0.57 (50.8, 0)
Urinary β2-microglobulin, mg/g creatinine	20.3 ± 9.1 (794, 0.17)
Urinary α1-microglobulin, mg/g creatinine	0.098 ± 0.018 (1.78, 0)

Values are expressed as mean ± SEM. eGFR, estimated glomerular filtration rate; SBP, systolic blood pressure; PAC, plasma aldosterone concentration; HDL, high-density lipoprotein; LDL, low-density lipoprotein; NAG, N-acetyl-β-D-glucosaminidase. Urinary markers are normalized by urinary creatinine concentration.

### Prospective 3-year observation of patients with CKD

**Figure 2:**
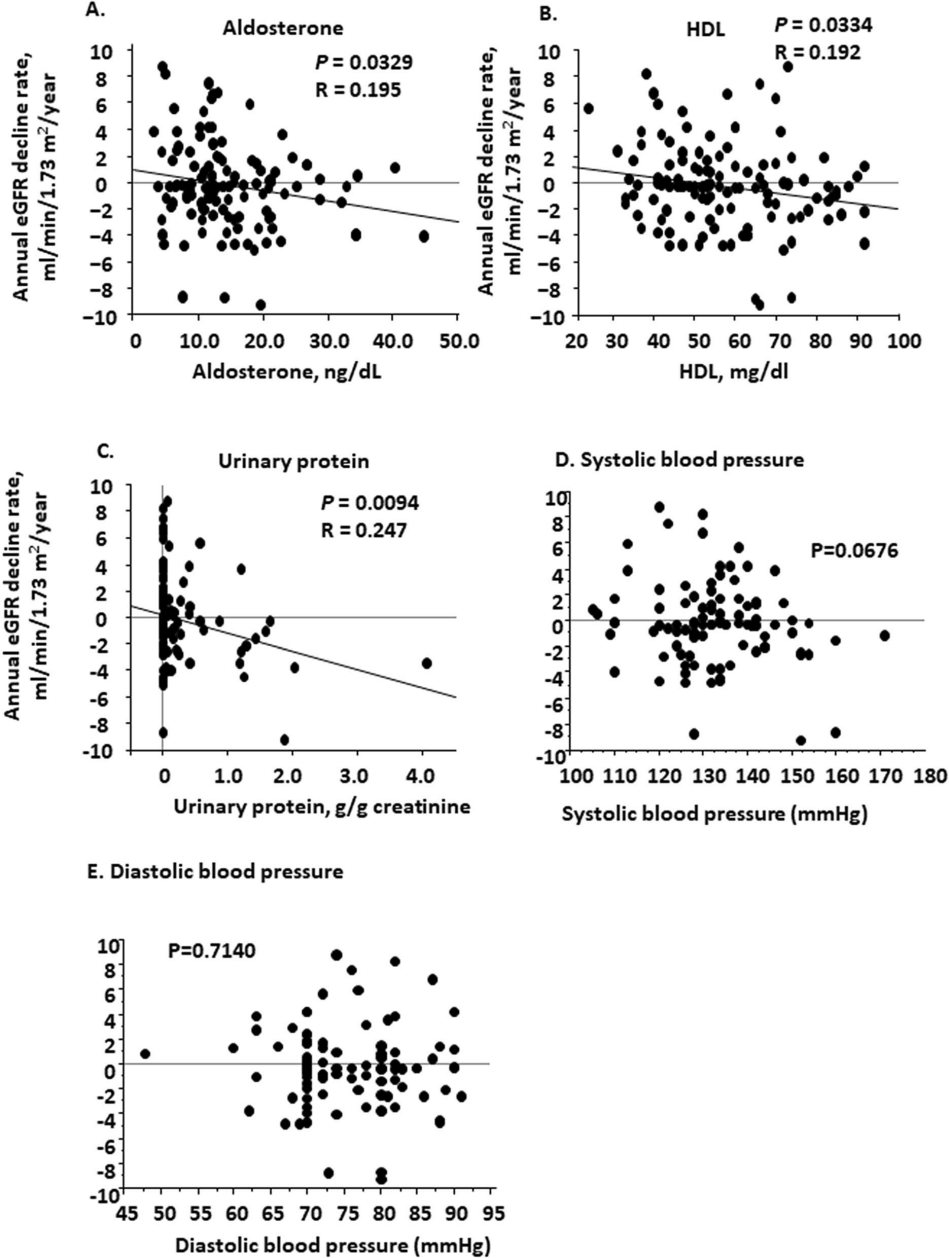
Linear regression analysis between annual eGFR change and baseline biochemical parameters in the observational study. After the 3-year observation of the cohort patients with CKD (n = 141), linear regression analysis showed a significant association between the annual eGFR change and plasma aldosterone concentration (**A**), plasma high-density lipoprotein concentration (**B**), urinary protein excretion (**C**), systolic blood pressure (**D**), and diastolic blood pressure (**E**). CKD, chronic kidney disease; eGFR, estimated glomerular filtration rate.

We prospectively observed these patients (151 patients) from 2007 through 2011 for 3 years. During this period, 10 patients were dropped from observation: five patients terminated clinic visits, two patients died, and three patients began hemodialysis. The remaining patients (141 patients) were subject to the analysis. Of 141 patients, 18 patients were diabetic. The average follow-up period was 3.04 ± 0.8 years. The mean eGFR changes were from 57.2 ± 1.64 ml/min/1.73 m^2^ to 58.0 ± 1.84 ml/min/1.73 m^2^ for the observation period, and the annual eGFR change in this CKD cohort was − 0.235 ± 0.294 ml/min/1.73 m^2^/year. The maximum annual increase was 8.71 ml/min/1.73 m^2^/year and maximal annual decline was − 9.32 ml/min/1.73 m^2^/year. Linear regression analysis revealed that the rate of change in eGFR of each patient was significantly correlated with his or her PAC, plasma HDL-cholesterol concentration, and urinary protein excretion at the baseline (Fig. 2, aldosterone, R = 0.195, *p* = 0.0329, HDL, R = 0.192, *p* = 0.00334,urinary protein, R = 0.247, *p* = 0.0094). We did not observe the significant relationship between the rate of change in eGFR of each patient and baseline SBP nor DBP (Fig. 2, SBP, *p* = 0.676; DBP, *p* = 0.7140). Multiple regression analysis using several clinical parameters at the baseline including age, eGFR, aldosterone, HDL-cholesterol, urinary NAG, urinary β2-microglobulin, α1-microglobulin, urinary albumin, and urinary protein levels demonstrated that PAC was independently associated with the rate of change in eGFR (Table 3, β = − 0.257, *p* = 0.0118). When we added SBP and DBP to the above nine factors in multiple regression analysis, no factors turned out to be independently associated with the rate of change in eGFR (Supplementary Table 1).

**Table 3:** Multiple regression analysis for the risk of the rate of change in eGFR in the observational study.

Parameter	β	*P* value
Age	0.002	0.9821
eGFR, ml/min/1.73 m^2^	− 0.125	0.2755
PAC, ng/dL	− 0.257	0.0118
Serum HDL-cholesterol, mg/dL	− 0.147	0.1302
Urinary NAG, IU/g creatinine	− 0.336	0.0497
Urinary β2-microglobulin, mg/g creatinine	− 0.122	0.2325
Urinary α1-microglobulin, mg/g creatinine	0.227	0.0878
Urinary albumin, mg/g creatinine	− 0.378	0.4714
Urinary protein, g/g creatinine	0.183	0.4122

Urinary markers are normalized by urinary creatinine concentration. eGFR, estimated glomerular filtration rate; PAC, plasma aldosterone concentration; HDL, high-density lipoprotein; NAG, N-acetyl-β-D-glucosaminidase.

**Figure 3:**
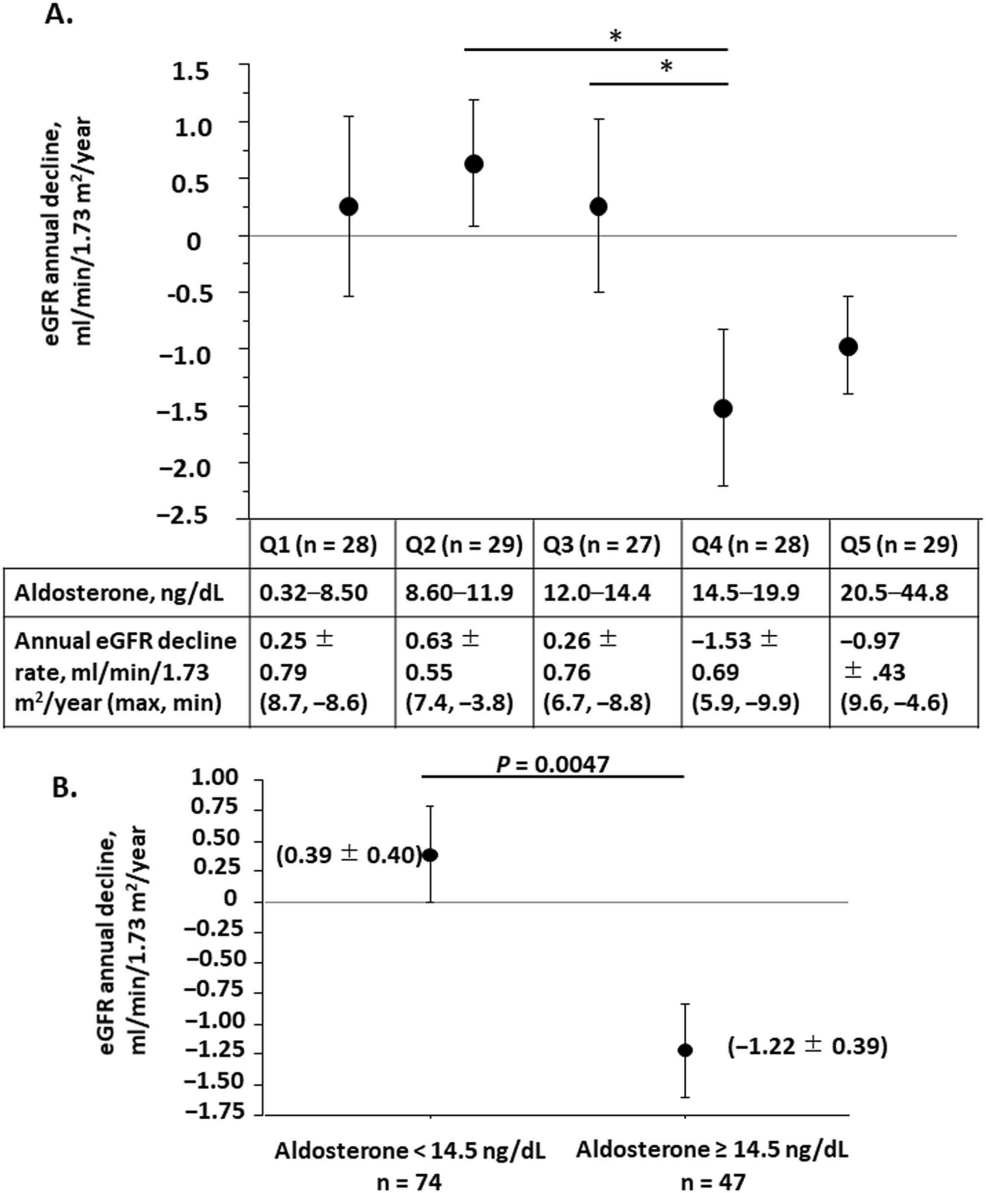
Annual eGFR change of CKD groups classified by baseline plasma aldosterone concentration in the observational study. (**A**) The CKD cohort was classified into five quintile groups according to the baseline plasma aldosterone concentration. The annual change of each group was compared among the groups by one-way analysis of variance. The lower table shows the plasma aldosterone range and annual change in eGFR of each group. (**B**) The CKD cohort was classified into the high aldosterone group (aldosterone ≥ 145 ng/dL) and low aldosterone group (aldosterone < 145 ng/dL). The annual change in each group was compared between the groups by unpaired t-test. Data are expressed as mean ± SEM. CKD, chronic kidney disease; eGFR, estimated glomerular filtration rate; SEM, standard error of the mean. *p < 0.05.

We further analyzed the relationship between baseline PAC and eGFR change. We classified patients into five quintile groups according to baseline PAC. The lowest PAC group (Q1) comprised 28 patients whose PAC was between 3.2 and 8.5 ng/dL. The second-lowest group (Q2) of 29 patients ranged between 8.6 and 11.9 ng/dL. The middle-range group (Q3) of 27 had aldosterone levels between 12.0 and 14.5 ng/dL. The second-highest group (Q4) of 28 patients ranged between 14.5 and 19.9 ng/dL. The highest group (Q5) of 29 patients ranged between 20.5 and 44.8 ng/dL (Table 4). Except for in gender, no significant differences were observed among the five groups in age, baseline eGFR, urinary protein excretion, and urinary albumin excretion (Table 4). The rate of change in eGFR of each group was 0.25 ± 0.79 ml/min/1.73 m^2^/year in the Q1 group, 0.63 ± 0.55 in Q2, 0.26 ± 0.76 in Q3, − 1.53 ± 0.69 in Q4, and − 0.97 ± 0.43 in Q5 (Fig. 3A). We compared these values and found that the rate of change in eGFR was significantly greater in the Q4 group than in the Q2 (*p* = 0.022) and Q3 (*p* = 0.048) groups. The Q4 group exhibited the fastest annual rate of change in eGFR among the five groups (Fig. 3A). The Q5 also showed the greater rate of change in eGFR as compared to the Q2 (*p* = 0.061) and Q3 (*p* = 0.076) groups, although the difference did not reach statistically significance (Fig. 3A). It appeared as a U-shape trend of the GFR change in relation to baseline PAC. Therefore, we set the cut-off value at 14.5 ng/dL and divided the patients into two groups. The annual rate of change in eGFR of the high aldosterone group (aldosterone ≥ 14.5 ng/dL) was − 1.22 ± 0.39 ml/min/1.73 m^2^ on average, which was greater than that of the low aldosterone group (aldosterone < 14.5 ng/dL), 0.39 ± 0.40 ml/min/1.73 m^2^/year on average (*p* = 0.0047, Fig. 3B).

**Table 4:** Patients with CKD grouped according to plasma aldosterone concentration in the observational study.

	Q1 n = 28	Q2 n = 29	Q3 n = 27	Q4 n = 28	Q5 n = 29	*P*
PAC, ng/dL	3.2–8.5	8.6–11.9	12.0–14.4	14.5–19.9	20.5–44.8	*P* < 0.0001
	6.20 ± 0.29	10.5 ± 0.20	13.0 ± 0.16	17.1 ± 0.32	26.7 ± 1.22	
Age	66.3 ± 2.6	64.8 ± 2.2	62.4 ± 2.8	59.8 ± 2.5	61.9 ± 3.1	*P* = 0.453
Gender, male/female	12/16	10/19	17/10	16/12	18/11	*P* = 0.0376
Baseline eGFR, ml/min/1.73 m^2^	62.2 ± 3.7	60.7 ± 3.2	59.3 ± 4.6	49.9 ± 3.1	54.0 ± 3.3	*P* = 0.098
Urinary protein, g/g creatinine	0.136 ± 0.065	0.192 ± 0.098	0.509 ± 0.281	0.526 ± 0.199	0.406 ± 0.163	*P* = 0.385
Urinary albumin, mg/g creatinine	83.1 ± 43.5	30.5 ± 10.4	34.3 ± 17.3	141.7 ± 57.1	126.8 ± 52.0	*P* = 0.168

Urinary markers are normalized by urinary creatinine concentration. Values are expressed as mean ± SEM

CKD, chronic kidney disease; eGFR, estimated glomerular filtration rate; PAC, plasma aldosterone concentration; Q, quartile; SEM, standard error of the mean.

### Long-term intervention study of EPL, the selective MRA

**Figure 4:**
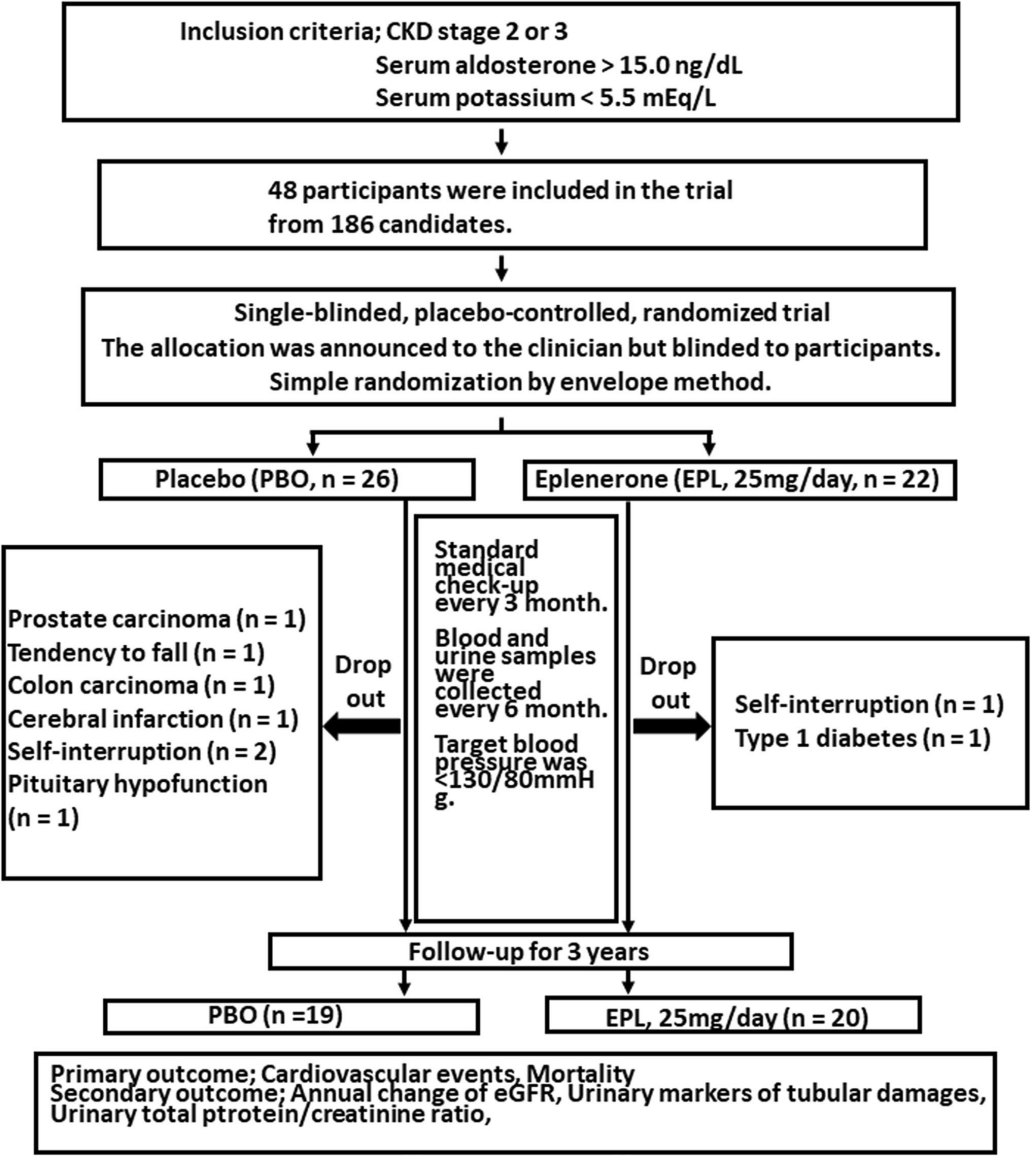
Protocols for patients allocated to EPL or PBO groups in the intervention study. Patients with CKD were randomly allocated to be treated with EPL (n = 22) or with PBO as the control group (n = 26).

To conduct the intervention study, we first made a list of CKD patients who had visited the renal division of Keio university hospital and fulfilled the diagnostic criteria of CKD as of June 2011. Among 186 patients with CKD, 133 candidates were excluded because their serum aldosterone concentration were less than 15.0 ng/dl or their serum potassium concentration were above 5.5 mEq/L. Finally, we recruited 48 patients who were eligible for this study during one-year recruitment period and allocated them to the EPL group (n = 22) or to the PBO control group (n = 26) using simple randomization by envelope method. During the study period, two patients in the EPL group and seven patients in the PBO group were dropped from the study (see Fig. 4). Ultimately, 20 patients in the EPL group and 19 patients in the PBO group completed the 3-year intervention.

**Figure 5:**
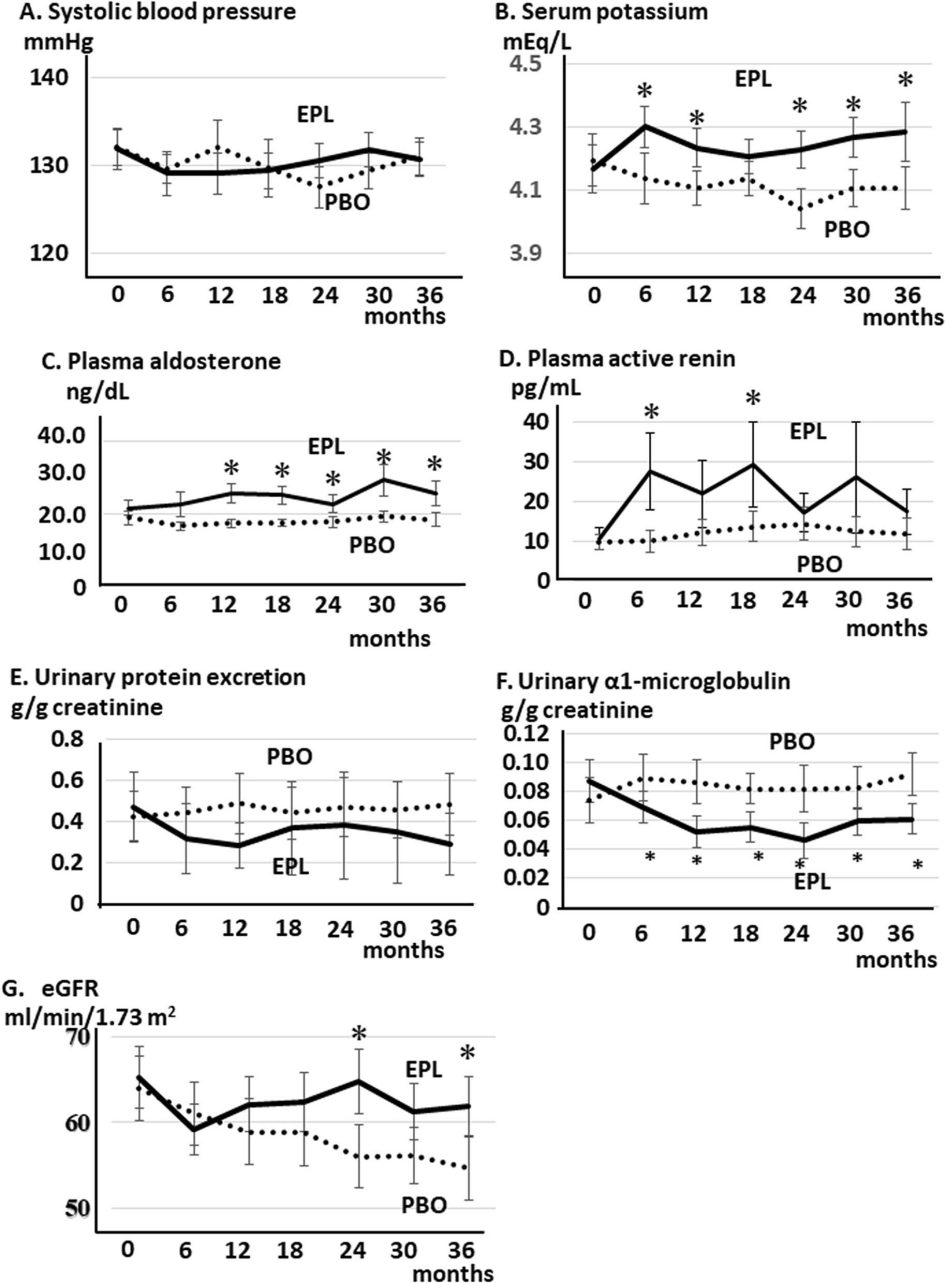
Line plot showing temporal changes in parameters of the EPL and PBO groups in the intervention study. (**A**) Systolic blood pressure, (**B**) serum potassium concentration, (**C**) plasma aldosterone concentration, (**D**) plasma active renin concentration, (**E**) urinary protein excretion, (**F**) urinary α1-microglobulin excretion, and (**G**) eGFR in each experimental group. The dotted line represents the data of the PBO group and the dashed line the data of the EPL group. Data are expressed as mean ± SEM. eGFR, estimated glomerular filtration rate; EPL, eplerenone; PBO, placebo; SEM, standard error of the mean. *p < 0.05 vs. the PBO group.

The baseline demographic and clinical characteristics of the EPL group and of the PBO group are shown in Table 5. The average of eGFR of two group are 63.9 ± 3.73 ml/min/1.73 m^2^ (PBO group) and 65.2 ± 3.59 ml/min/1.73 m^2^ (EPL group), respectively. Any of the parameters examined was not different between the two groups. The etiology of CKD was similar. The major cause of CKD in each group was nephrosclerosis and almost 90% of patients in each group were non-diabetic. The antihypertensive medications were not different between the two groups. Mean serum potassium concentration of around 4.2 mEq/L in each group is much higher than the mean value of 3.7 mEq/L in the observational study. It may be because the intervention study had a greater percentage of patients on ACEI/ARB compared to the observational study. We continued this protocol for 3 years and compared the various clinical parameters between the groups every 6 months (Fig. 4). There were no patients whose ARBs or ACE inhibitors were discontinued during the study period. EPL treatment at the dose of 25 mg had no effect on SBP levels throughout the study period (Fig. 5A, PBO group: 0 month, 132.1 ± 2.04 mmHg; 6 month,129.6 ± 1.68 mmHg; 12 month, 132.1 ± 3.01 mmHg; 24 month, 127.5 ± 2.35 mmHg; 36 month,130.9 ± 2.15 mmHg; EPL group: 0 month, 131.9 ± 2.31 mmHg; 6 month, 129.1 ± 2.54 mmHg; 12 month, 129.1 ± 2.30 mmHg; 24 month, 130.5 ± 1.95 mmHg; 36 month, 130.7 ± 1.86 mmHg). Plasma potassium concentration in EPL group increased significantly as compared with that in PBO group (EPL group, 4.32 ± 0.07 mEq/L; PBO group, 4.14 ± 0.08 mEq/L) as early as 6 months after the initiation of the study and was sustained through the study period. However, the maximum concentration reached 4.9 mEq/L in one participant in the EPL group at 36 months, and we did not observe a serious hyperkalemic episode during the course of the study (Fig. 5B, PBO group: 0 month, 4.19 ± 0.08 mEq/L; 6 month, 4.14 ± 0.08 mEq/L; 12 month, 4.11 ± 0.05 mEq/L; 24 month, 4.04 ± 0.06 mEq/L; 36 month, 4.12 ± 0.07 mEq/L; EPL group: 0 month, 4.17 ± 0.078 mEq/L; 6 month, 4.30 ± 0.07 mEq/L; 12 month, 4.23 ± 0.07 mEq/L; 24 month, 4.22 ± 0.07 mEq/L; 36 month, 4.29 ± 0.09 mEq/L). PAC significantly increased as early as month 12 in the EPL group as compared with that in the PBO group. The increase observed in the EPL was sustained through the study period (Fig. 5C, PBO group: 0 month, 18.9 ± 1.86 ng/dL; 6 month, 18.9 ± 1.11 ng/dL; 12 month, 16.7 ± 1.08 ng/dL; 24 month, 1.76 ± 1.51 ng/dL; 36 month, 19.3 ± 1.91 ng/dL; EPL group: 0 month, 21.6 ± 2.16 ng/dL; 6 month, 22.6 ± 3.32 ng/dL; 12 month, 25.7 ± 2.74 ng/dL; 24 month, 22.8 ± 2.55 ng/dL; 36 month, 25.7 ± 3.50 ng/dL). Plasma active renin concentration increased by EPL treatment and the concentrations at month 6 and 18 in EPL was greater than those in PBO (*p* < 0.05) (Fig. 5D, PBO group: 0 month, 9.86 ± 1.97 pg/mL; 6 month, 10.0 ± 2.62 pg/mL; 12 month, 12.3 ± 3.32 pg/mL; 18 month, 13.8 ± 3.75 pg/mL; 24 month, 14.5 ± 4.20 pg/mL; 36 month, 11.8 ± 3.96 pg/mL; EPL group: 0 month, 10.7 ± 2.67 pg/mL; 6 month, 27.6 ± 9.57 pg/mL; 12 month, 21.9 ± 8.49 pg/mL; 18 month, 29.3 ± 10.7 pg/mL; 24 month, 17.2 ± 4.79 pg/mL; 36 month, 17.4 ± 5.7 pg/mL). Urine protein to creatinine ratio (g/g creatinine) dropped as early as 6 month in the EPL group, and its levels remained lower in the EPL group than in the PBO group, although the differences were not statistically significant (Fig. 5E, PBO group: 0 month, 0.42 ± 0.12; 6 month, 0.44 ± 0.12; 12 month, 0.49 ± 0.15; 24 month, 0.47 ± 0.14; 36 month, 0.48 ± 0.15; EPL group: 0 month, 0.47 ± 0.16; 6 month, 0.32 ± 0.17; 12 month, 0.28 ± 0.11; 24 month, 0.38 ± 0.26; 36 month, 0.29 ± 0.15). Urine α1-microglobulin to creatinine ratio (g/g creatinine), a urinary marker for tubular damage, significantly decreased as early as month 6 in the EPL group as compared with that in the PBO group. The decrease observed in the EPL was sustained through the study period (Fig. 5F, PBO group: 0 month, 7.41 ± 1.57; 6 month, 8.96 ± 1.64; 12 month, 8.67 ± 1.51; 24 month, 8.17 ± 1.61; 36 month, 9.18 ± 1.49; EPL group: 0 month, 8.72 ± 1.48; 6 month, 6.94 ± 1.09; 12 month, 5.22 ± 1.10; 24 month, 4.63 ± 1.22; 36 month, 6.10 ± 1.03). After first 6 months of EPL treatment, the eGFR decreased from 65.21 ± 3.59 ml/min/1.73 m^2^ to 59.11 ± 3.00 ml/min/1.73 m^2^, and the levels were lower in the EPL group than in the PBO group (Fig. 5G, from 63.95 ± 3.73 ml/min/1.73 m^2^ to 60.98 ± 3.65 ml/min/1.73 m^2^). Thereafter, the eGFR remained stable, and at the end of 36 months of treatment, the eGFR was 61.86 ± 3.50 ml/min/1.73 m^2^. The annual decline rate was -1.117 ml/min/1.73m^2^/year. In contrast, the eGFR declined slowly but progressively for 3 years in the PBO group at the rate of − 3.108 ml/min/1.73 m^2^/year (baseline 63.95 ± 3.73 ml/min/1.73 m^2^, 36 months, 54.62 ± 3.67 ml/min/1.73 m^2^). The eGFR levels were significantly higher in the EPL group than in the PBO group at month 24 and month 36 in the study period. The eGFR levels at month 24 in EPL was 64.67 ± 3.76 ml/min/1.73 m^2^, which was greater than those in PBO, 55.99 ± 3.74 ml/min/1.73 m^2^ (*p* < 0.05, Fig. 5G). The eGFR levels at month 36 in EPL was 61.86 ± 3.50 ml/min/1.73 m^2^, which was also greater than those in PBO, 54.62 ± 3.67 ml/min/1.73 m^2^ (*p* < 0.05, Fig. 5F). EPL inhibited the decline in eGFR and maintained renal function without any changes in blood pressure or urinary protein excretion, indicating renoprotective effects by EPL independent of its effects on proteinuria or blood pressure.

**Table 5:** Baseline characteristics of the EPL and PBO groups in the intervention study.

Parameter	Placebo (n = 19)	Eplerenone (n = 20)	*P*
SBP, mmHg	132.1 ± 2.04	131.9 ± 2.31	0.886
DBP, mmHg	76.3 ± 1.07	76.6 ± 1.46	0.509
eGFR, ml/min/1.73 m^2^	63.9 ± 3.73	65.2 ± 3.59	0.598
Serum creatinine, mg/dL	0.91 ± 0.07	0.88 ± 0.06	0.610
Fasting blood sugar, mg/dL	109.2 ± 3.1	111.7 ± 3.50	0.409
IRI, μU/mL	12.1 ± 0.4	12.6 ± 3.0	0.642
Glycated albumin, %	14.4 ± 0.36	15.0 ± 0.30	0.243
PAC, ng/dL	18.9 ± 1.86	21.6 ± 2.16	0.315
Plasma active renin, pg/mL	9.86 ± 1.97	10.7 ± 2.67	0.798
Serum potassium, mEq/L	4.19 ± 0.08	4.17 ± 0.08	0.826
Urine α1-microglobulin/creatinine, mg/g	7.41 ± 1.57	8.72 ± 1.48	0.354
Urine NAG/creatinine, IU/g	7.25 ± 0.77	6.68 ± 0.88	0.373
Urine β2-microglobulin/creatinine, mg/g	144.2 ± 22.8	262.0 ± 86.3	0.227
Urine protein/creatinine, g/g	0.42 ± 0.12	0.47 ± 0.16	0.813
**Cause of CKD**			
Nephrosclerosis (%)	11/19 (57.9%)	14/20 (70.0%)	0.515
Chronic glomerulonephritis (%)	5/19 (26.3%)	5/20 (25.0%)	0.925
Diabetic nephropathy (%)	2/19 (10.5%)	1/20 (5.0%)	0.605
Others (%)	1/19 (5.3%)	0/20 (0%)	0.482
**Antihypertensive medication**			
ARB or ACEI (%)	15/19 (78.9%)	14/20 (70.0%)	0.716
Calcium channel blocker (%)	12/19 (63.2%)	9/20 (45.0%)	0.341
Diuretic (%)	5/19 (26.3%)	3/20 (15.0%)	0.451
β-blocker (%)	2/19 (10.5%)	4/20 (20.0%)	0.661

Urinary markers are normalized by urinary creatinine concentration. Values are expressed as mean ± SEM. ACE; angiotensin converting enzyme.

SBP, systolic blood pressure; ACEI, angiotensin converting enzyme inhibitor; ARB, angiotensin II receptor blocker; eGFR, estimated glomerular filtration rate; EPL, eplerenone; PAC, plasma aldosterone concentration; NAG, N-acetyl-β-D-glucosaminidase; PBO, placebo; SEM, standard error of the mean.

## Discussion

In the cross-sectional observational study of the present study, we found that in patients with CKD, the baseline eGFR levels were associated with plasma aldosterone levels, and the annual change in eGFR was also independently associated with plasma aldosterone levels. When we divided the patients into the five quintile groups according to baseline PAC, the fourth quintile (PAC, 14.5–19.9 ng/dL) exhibited the fastest annual eGFR change. In the intervention trial of the present study, we examined the effect of treatment with MRA on the progression of renal damage. We used data obtained in the prior cross-sectional observational study to treat patients with CKD who had high aldosterone levels (PAC > 15.0 ng/dL) with an MRA (EPL at 25 mg) or with a PBO as a control and examined its effects on CKD progression as well as various urinary markers for renal damage. Although blood pressure and urinary protein excretion were not altered significantly, EPL significantly sustained eGFR levels for as long as 3 years as compared with the PBO. EPL had no significant effects on blood pressure levels. Our observation is consistent with the recent meta-analysis which shows EPL at a dose of 25 mg/day did not produce a statistically significant reduction in systolic or diastolic blood pressure^18^. Our data indicated that MRA maintain eGFR independently of its effects on blood pressure. Moreover, EPL reduced the α1-microglobulin levels through the entire intervention period. Plasma potassium levels were significantly increased by EPL treatment up to 4.9 mEq/L at the maximum in one patient, although serious adverse events were not documented. We suggest that high plasma aldosterone might be a risk factor for the progression of renal impairment. For patients with CKD who had a high aldosterone level above 15.0 ng/dL we demonstrated that MRA exerted the renal protective effects independent of its effect on blood pressure.

Aldosterone is a reported risk factor for cardiovascular disease, although its role in kidney damage has not been well established. As we observed in CKD cohort, CKD is characterized by elevated levels of aldosterone, and this fact has been demonstrated both in animals^19^ and humans^20,21^. A causal role of aldosterone in renal impairment was demonstrated by an animal study. In 5/6th nephrectomized rats, renal impairment was less pronounced when rats were adrenalectomized at the same time^22^. The unfavorable effects by aldosterone were also reported in human study previously where serum aldosterone levels were associated with renal dysfunction and renal tissue scarring proven by renal biopsy^23^. These effects were reported to be through the activation of MR leading to tubulointerstitial inflammation and fibrosis^24^. Aldosterone is also reported to elevate renal vascular resistance and glomerular capillary pressure, which also contributes to proteinuria and renal tissue damages^25^. Our CKD cohort data also supported this effect in that PAC was positively correlated with proteinuria, albuminuria, and urinary excretion of renal tubular damage markers. By multiple regression analysis, we further found that plasma aldosterone level was an independent risk factor for future eGFR decline in addition to basal urinary protein excretion. Our patients were already treated with various renoprotective reagents including ARBs or ACEIs. In this situation, MR activation can be a persistent and untreated risk factor that affects renal function.

Several small short-term clinical studies have examined the effects of adding MR blockers to ACEIs and/or ARBs in proteinuric kidney disease^26^. Most patients in these studies had diabetes, hence, the best evidence for using aldosterone blockade in proteinuric CKD was in the treatment of diabetic nephropathy^27–29^. Recently, FIDELIO-DKD, a randomized, double-blind, placebo-controlled, parallel-group, event-driven trial using finerenone, a novel, non-steroidal, selective mineralocorticoid-receptor antagonist was reported and the participants of the study was composed of diabetic 5,734 patients^15^. In contrast, the data supporting the use of MRA in nondiabetic kidney disease are not as robust as those in diabetic nephropathy^30,31^. These studies demonstrated mainly antiproteinuric effects by MRAs, and the effects on eGFR were still inconsistent. This effect of maintenance of eGFR levels was documented in a previous report in which spironolactone was administered to patients with CKD already treated with ACEIs or ARBs^21^. However, there are also reports in which MRAs had no effect on renal function during the study period^27,32^. Recent meta-analyses on the renal effects by MRAs have also revealed that MRA therapy added to ACEIs or ARBs significantly reduced daily urinary protein excretion compared with ACEIs or ARBs alone, while it did not significantly affect eGFR levels^14,33^. The results of FIDELIO-DKD supports the renoprotective effects by MRA^16^ which was consistent with our results of the intervention study. This inconsistent outcome implied that MRA therapy may offer the most favorable effects on renal function in selected patients with CKD. We hypothesized that increased plasma aldosterone levels above 14.5 ng/dL or 15.0 ng/dL is one of the candidates to successful treatment with MRAs in patients with CKD. Another characteristic of our intervention study was that the participants were a heterogeneous group that comprised patients with CKD patients mainly having renal atherosclerosis. Almost 90% of the patients in each group were non-diabetic (Table 5). The present intervention study showed that even in patients with CKD who are not diabetic, irrespective of the pretreatment with ACEIs or ARBs, some of the patients were responsive to MRA treatment. We assume that these patients have high plasma aldosterone levels and proposed a cut-off value of 14.5–15.0 ng/dL.

The mechanisms for the maintenance of eGFR by the MRA in this study are not fully elucidated. The previous study also documented this effect with the initial drop in eGFR^21^. This phenomenon is reminiscent of the initial fall in eGFR seen in patients with CKD treated with ACEIs, whereby an initial rapid decrease in GFR is usually followed by stabilization of kidney function^34^. Although it was a nonsignificant decline, we also observed the initial fall in eGFR levels and following maintenance of eGFR. It was reported that aldosterone exerts rapid nongenomic effects on renal vasculature resulting in increased renal vascular resistance and reduced GFR in normal healthy volunteers^35^. The effects of aldosterone on renal vasculature occurred only when endothelial nitric oxide (NO) synthase was inhibited by simultaneous administration of the NO inhibitor, NG monomethyl-L-arginine, indicating that this vasoconstriction action might result in reduced renal blood flow^36^. Another report demonstrated that endothelial denudation and pharmacological blockade of endothelial nitric oxide synthase (eNOS) increased the sensitivity of afferent arterioles to aldosterone, suggesting that NO modulates the vasoconstrictor action of aldosterone^36^. Aldosterone downregulates eNOS expression levels^37^, which inferred endothelial dysfunction of the renal vasculature. Therefore, MRAs might ameliorate aldosterone-induced endothelial dysfunction by the restoration of eNOS activity^38^. Taken together, under the situation of endothelial dysfunction, including CKD, the vasoconstrictor action by aldosterone is more prominent in the efferent arterioles and maintains the GFR^25,35,36^. MRAs may increase NO availability and ameliorated endothelial dysfunction by aldosterone, which inactivated the vasoconstrictor effects of aldosterone on the efferent arteriole, which decreased the GFR. This, in turn, reduced the glomerular damage and exhibited long-standing eGFR maintenance effects. Although we did not measure endothelium-dependent vasodilation, in the present study, we recruited patients with CKD who had high levels of aldosterone that were suspected to have endothelial dysfunction. For these specific patient group, a clear renoprotective effect by the MRA could be observed.

The present intervention study also revealed that the MRA reduced the urinary excretion of α1-microglobulin, which implied that the MRA attenuated the proximal tubular cell damage in patients with CKD. The expression of the MR in the thick ascending limb, distal tubules, and cortical collecting duct (but not in the proximal tubules) of Wistar rats and mice has been demonstrated both by the polymerase chain reaction and by immunohistochemistry^39^. In contrast, other studies demonstrated the expression of MR mRNA in the S3 segments of the proximal tubules in rats^40,41^. The immunohistochemical demonstration of MR expression in human, rat, and mouse kidney proximal tubules has also been reported^42^. Aldosterone induces proximal tubular damage through increased oxidative stress^43^. Therefore, the reduction of α1-microglobulin implied that MRA might protect against the tubular damage by aldosterone. It is also assumed that the amelioration of renal vascular resistance and the vasodilator action on the efferent arterioles might improve the blood supply to the post-glomerular and peritubular capillaries around the proximal tubular cells that contributed to the reduction in urinary α1-microglobulin excretion. It has been shown that MRA blocks the profibrotic effects by aldosterone and ameliorates tubulointerstitial fibrosis through its effects on peritubular arterioles or interstitial fibroblasts^44^. These hemodynamic action, protective role in proximal tubular cell damages and anti-fibrotic effects might in combination contributed to stabilization of the renal function. Our data reinforced the previous clinical trials demonstrating the renoprotective effects by MRA^45,46^.

Despite relevant information obtained, our intervention study has several limitations. First, the sample size was so small that the study lacked the power to detect the difference in the primary outcome of cardiovascular events. We experienced only one event of cerebral infarction in the placebo group. Secondly, the study was conducted in one clinical center. Finally, the intervention study was single-blinded and the doctor who prescribed the medicine was aware whether the patient was allocated to PBO or EPL.

In conclusion, the cross-sectional observational study of the present study demonstrated that plasma aldosterone level was an independent risk factor for the progression of renal dysfunction and that MRA could reduce the rate of decline in eGFR in patients with CKD who had high PAC. Of note, we provided this evidence by the long-term clinical PBO-controlled clinical trial of EPL for 3 years. We found the maintenance of eGFR by EPL independent of its effects on proteinuria or blood pressure. We hypothesize that the patients with CKD with high plasma aldosterone levels are good candidates for treatment with an MRA such as EPL.

## Methods

### Patients

From April 2007 through July 2007, we recruited consecutive patients with CKD from the patients referred to the renal division of our department. Enrollees were diagnosed with CKD according to either one of the following criteria: (1) eGFR below 60 ml/min/1.73 m^2^, or (2) kidney damage evident from dipstick-detected urinary protein excretion for more than 3 months. Patients undergoing hemo- or peritoneal dialysis were excluded from this study. We prospectively observed the enrolled patients for 3 years during the 2007 to 2011 period. The main objective of this observation was to evaluate the long-term effects of various biochemical or physiological parameters on the progression of renal dysfunction. Patients who began dialysis during the observation period were excluded in the analysis. Biochemical data were collected when the patients were recruited, 3 months after the recruitment, and when the observation period was finished. Each patient visited our clinic every 3 months and was treated for renal dysfunction with optimal medical intervention. All the studies were conducted after obtaining informed consent from each patient by opt-out fashion. To conduct the clinical study, the authors adhered to Declaration of Helsinki. This study protocol was approved by the Ethical Committee of Keio University. Three months after the first visit, each patient provided informed consent, and the 3-year observation period began.

### Placebo-control intervention parallel study with EPL

From our prior observations, we hypothesized that high PAC plays a pivotal role in the forward decline in renal function through its deteriorative effects on the kidney. We supposed that those patients would acquire clinical benefit from the treatment with MRA. To demonstrate this hypothesis, we conducted a placebo-control, randomized, parallel-grouped study using EPL. We recruited the patients with CKD who had high aldosterone levels (above 15.0 ng/dL) and treated them with selective MRA, eplerenone (EPL) or placebo (PBO). After the one-month observation period, we conducted the intervention study to eligible patients from our cohort using aldosterone blockade with EPL. The inclusion criteria were: (1) CKD, stage 2 or stage 3, (2) PAC was above 15.0 ng/dL (the cut-off value determined from our first-phase observation that patients with CKD and PAC was above 14.5 ng/dL had the worst prognosis in terms of renal deterioration), (3) serum potassium level was below 5.5 mEq/L. This is an exploratory intervention study and at first, we determined the sample size as forty CKD patients in each group. The inclusion period was first set at one year and the follow-up period at three years. These plans in the protocol were defined on January 11, 2011. The protocol was fixed and approved by the Ethical Committee of Keio University in July 2011. The recruit started in August 2011 and had planned to end in June 2012. However, since the participants number turned out too small to reach the expected number, we extended the deadline until December 2012. We first made a list of 186 CKD patients who fulfilled the diagnostic criteria of CKD as described above as of June 2011. After this registration, we enrolled the eligible patients who met the inclusion criteria one by one in order of his or her hospital visit. The patient inclusion and the data collection were performed at the department of medicine, Keio University Hospital. After obtaining the patients’ consent, we randomly allocated patients eligible for this study to the EPL group or the PBO control group by envelop method. The PBO was created similarly in size and shape as eplerenone tablet. One month after the registration, each patient took EPL or PBO. The dosage of EPL was fixed at 25 mg/day throughout the study period. The date of the first registration was on August 2, 2011 and the date of the last registration was on December 20, 2012. The date of study registration 48 registrations composed of 26 PBO-treated and 22 EPL-treated participants were recruited in the study. Due to this randomization, the number of each group had become unequal. The allocation was announced to the clinicians although the patients were blinded to which group they were allocated. One investigator (HM) had the responsibility of preparing the envelopes and random allocation. For the safety of the study, the patients visited the clinic of department of medicine in Keio University hospital every 3 months for regular check-ups when the clinical data were obtained. The blood and urine samples were obtained every 6 months. If the patients’ serum potassium levels were over 5.5 mEq/L at any time during the study period, it was determined that the participants should be excluded from the study. The both groups received EPL or PBO in addition to the ongoing conventional treatment. During the intervention period, baseline doses of ACEI, ARB, or both were not changed at the beginning of study. Subsequently, we planned to modify these medications according to blood pressure and serum potassium levels although no patients had their ACEI/ARB discontinued. For adequate control of serum potassium, we advised a low potassium diet to all patients. The parallel two groups were followed for 3 years and follow-up ended in January 2016 as defined in the original study design. As described in Fig. 4, among 26 PBO-treated participants, 7 were dropped out from the study during the 3-year study period. The reasons for the withdrawal were prostate carcinoma, poor health status, tendency to fall, colon carcinoma, cerebral infarction, self-interruption, and pituitary hypofunction. Among 22 EPL-treated participants, 2 participants were dropped out because of poor health status and development of type 1 diabetes. Finally, 19 PBO-treated participants and 20 EPL-treated participants were subject to the analysis. The primary outcome of this intervention was HOMA-IR, rate of change in eGFR, incidence of CVD, mortality rate and secondary outcomes were the changes in various renal parameters including the change in urinary protein excretion, urinary α1-microglobulin. As described, we first set the incidence of cardiovascular event and mortality as primary outcomes, although the power of the sample size is too small (placebo group, n = 26, eplerenone group, n = 22) to evaluate the results because of the short inclusion period. However, we obtained some effects on the progression of CKD evaluated as eGFR decline and urinary protein excretion as one of the primary endpoint and secondary outcome, which can be described as the changes in renal parameters by the intervention. This study protocol was approved by the Ethical Committee of Keio University on July 1, 2011 and registered to UMIN000008521 on July 24, 2012. All the studies were conducted after obtaining the written informed consent from each patient.

### Blood and urine samples

All samples were taken for the measurement of routine chemistry after overnight fasting, which includes serum concentrations of potassium, creatinine, glucose, glycated albumin, total cholesterol, low-density lipoprotein (LDL) cholesterol, high-density lipoprotein (HDL) cholesterol, and triglyceride. We also measured urinary concentrations of *α*1-microglobulin, *β*2-microglobulin, protein, albumin, and creatinine in addition to urinary activity of N-acetyl-β-D-glucosaminidase (NAG). GFR was estimated by modified the Modification of Diet in Renal Disease (MDRD) Study equation adapted for the Japanese population: eGFR = 194 ×  (serum creatinine in mg/dL) ^−1.094^ × age^−0.287^ ×  (0.739 for women)^47^. Plasma aldosterone and cortisol were measured by radioimmunoassay kit (Mitsubishi Chemical Medicine, Tokyo, Japan).

### Statistical analysis

Continuous variables were expressed as mean ± standard error and categorical variables as proportions or ratios. We used the Student’s t-test or non- Student’s t-test and the Mann–Whitney U-test for the comparison of non-parametric parameters. We used univariate analyses to assess correlations between various baseline parameters in our prospective observational study. In the multiple linear regression analyses for the identification of risk factors for a change in eGFR, we selected any covariates that were significant in univariate analysis as well as baseline age, eGFR, and urinary albumin excretion. In the comparison among the five quintile groups classified by baseline PAC, we used one-way analysis of variance as appropriate, followed by Bonferroni’s post hoc test. In the intervention study, the comparison between the EPL group and placebo group was performed by Wilcoxon signed-ranks test. The statistical analyses were performed using StatView J-4.5 statistical software (Abacus Concepts, Berkeley, CA, USA). A *P* value < 0.05 was considered significant.

## Supplementary information

Supplementary Information.
