# Supplementary material for: The effect of aldosterone and aldosterone blockade on the progression of chronic kidney disease: a randomized placebo-controlled clinical trial
Source: Sci Rep. 2020 Oct 6;10:16626. doi: 10.1038/s41598-020-73638-4 (PMC7538950; doi:10.1038/s41598-020-73638-4)
Supplement: Supplementary file 1 — Supplementary Information. [file 41598_2020_73638_MOESM1_ESM.pdf]

Supplementary Information

The effect of aldosterone and aldosterone blockade on the progression of chronic kidney disease: a randomized placebo-controlled clinical trial

Hitoshi Minakuchi, Shu Wakino, Hidenori Urai, Arata Kurokochi,

Kazuhiro Hasegawa, Takeshi Kanda, Hirobumi Tokuyama, and Hiroshi Itoh

Supplementary Table 1. Multiple regression analysis for the risk of eGFR annual change in the observational study.

Parameter	β	*P*-value
Age	0.032	0.8068
eGFR, ml/min/1.73 m^2	−0.054	0.6823
Aldosterone, pg/ml	−0.206	0.0825
HDL-cholesterol, mg/dl	−0.147	0.1951
Urinary NAG, IU/g creatinine	−0.341	0.1164
Urinary β2-microglobulin, mg/g creatinine	−0.142	0.2487
Urinary α1-microglobulin, mg/g creatinine	0.252	0.1040
Urinary albumin, mg/g creatinine	−0.306	0.2311
Urinary protein, g/g creatinine	0.118	0.6876
Systolic blood pressure	-0.218	0.996
Diastolic blood pressure	0.111	0.4049

Values are expressed as mean ± SEM. eGFR, estimated glomerular filtration rate; HDL, high-density lipoprotein; HOMA-IR, Homeostasis Model Assessment of Insulin Resistance; LDL, low-density lipoprotein; NAG, N-acetyl-β-D-glucosaminidase. Urinary markers are normalized by urinary creatinine concentration.

Supplementary Figure 1, Relationship of blood pressure to eGFR and plasma aldosterone concentration in the cross-section study.

(A) Linear regression analyses were performed between eGFR and systolic blood pressure or diastolic blood pressure. (B) Linear regression analyses were performed between plasma aldosterone concentration and systolic blood pressure or diastolic blood pressure. eGFR, estimated glomerular filtration rate.

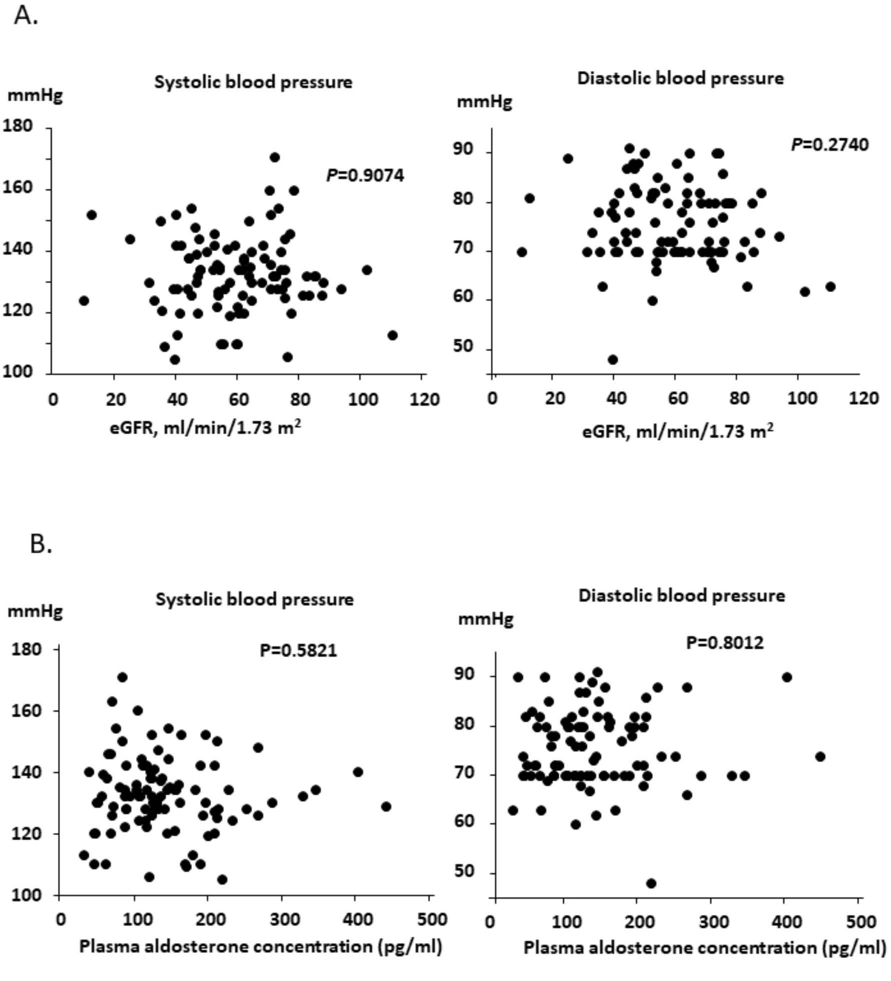

Supplementary Figure 1, Minakuchi, et al.
